# Causes of long-term mortality in patients with ST-segment elevation myocardial infarction is dictated by the presence of microvascular obstruction

**DOI:** 10.1093/ehjopen/oeaf002

**Published:** 2025-01-23

**Authors:** Giselle Fisher, Brynn Okeson, Evan Walser-Kuntz, Joao L Cavalcante, Jay H Traverse

**Affiliations:** Minneapolis Heart Institute Foundation at Abbott Northwestern Hospital, 920 East 28th Street, Suite 300, Minneapolis, MN 55407, USA; University of Pennsylvania School of Medicine, 3400 Civic Center Blvd, Philadelphia, PA 19104, USA; Minneapolis Heart Institute Foundation at Abbott Northwestern Hospital, 920 East 28th Street, Suite 300, Minneapolis, MN 55407, USA; Minneapolis Heart Institute Foundation at Abbott Northwestern Hospital, 920 East 28th Street, Suite 300, Minneapolis, MN 55407, USA; Minneapolis Heart Institute Foundation at Abbott Northwestern Hospital, 920 East 28th Street, Suite 300, Minneapolis, MN 55407, USA; Minneapolis Heart Institute Foundation at Abbott Northwestern Hospital, 920 East 28th Street, Suite 300, Minneapolis, MN 55407, USA; Cardiovascular Division, University of Minnesota School of Medicine, 420 Delaware St SE MMC508, Minneapolis, MN 55455, USA

## Introduction

Microvascular obstruction (MVO) is observed in 40–60% of patients with ST-segment elevation myocardial infarction (STEMI) and is associated with adverse left-ventricular remodelling, heart failure, and increased mortality despite comprising only a small percentage of infarct size.^1–3^ Microvascular obstruction results from both intravascular and extravascular factors including embolization of plaque debris, vasoconstriction, microvascular dysfunction, and compressive myocardial oedema.^4–6^ However, the long-term effects of MVO on mortality and its causes have rarely been described. This analysis presents the longest-term follow-up to date of a cohort of STEMI patients with MVO from a single institution that describes their longevity and primary cause of death, which could provide insight into why MVO is associated with such high morbidity, despite its relatively small contribution to infarct size.

## Methods

This was a retrospective review of 475 STEMI patients (average age = 60 years, 76% male) who received successful reperfusion with primary percutaneous coronary intervention (PCI) between 2007 and 2017. All patients had cardiac MRI prior to discharge. Cardiac MRI imaging was assessed with a 1.5 T Scanner (Avanto, VCardiac MR Scanner, Siemens Medical Solutions, Malvern, PA), and MVO was measured 2 min following gadolinium administration (0.2 mmol/kg) using single-shot True-FISP short-axis imaging covering the left ventricle acquired in a single breath hold. Microvascular obstruction was identified as a hypo-enhanced signal within the delayed hyper-enhanced infarct region.

Causes of death were determined from the patient’s terminal hospitalization electronic medical record or death certificate when the death occurred at an outside facility. The study was approved by the Institutional Review Board of Allina Health. All patients signed written, informed consent to allow their data to be used for research purposes. All investigations conformed to the principles outlined in the Declaration of Helsinki.

## Results

Microvascular obstruction was observed in 72% of patients on cardiac MRI performed a median of 2 days (IQR = 1.0, 3.0) following STEMI. There was no difference between baseline demographics, cardiac risk factors, or previous cardiac procedures between the two groups (*Table 1*). The infarct-related artery was more frequently the LAD in patients with MVO who had greater ischaemic times (159 vs. 140 min; *P* = 0.011) and infarct size (*P* < 0.001) and were more likely to have TIMI 0 flow on presentation (71% vs. 54%; *P* < 0.003). Following PCI, there was a similar proportion of TIMI 3 flow (95% vs. 96%) between the two groups but the MVO group had a significantly higher residual ST-elevation on electrocardiogram and reduced LVEF (48% vs. 58%; *P* < 0.001) and larger LV volumes. Infarct size was significantly larger in those patients with MVO (44 ± 28 vs. 27.6 ± 20 g; *P* < 0.01) who had a mean MVO mass of 16.2 ± 19.2 g. A similar percentage (36%) of patients in both groups received glycoprotein IIB IIIA inhibitors during PCI.

**Table 1:** Baseline demographics, risk factors, cardiac MRI measurements, and laboratory findings

	MVO			
Variable	Overall, *n* = 492	Present, 353 (72%)	Not present, 139 (28%)	*P*-value
Baseline demographics				
Age, years, median (IQR)	60 (53, 68)	60 (52, 68)	60 (53, 68)	0.82^a^
Male, *n* (%)	372 (76%)	273 (77%)	99 (71%)	0.16^b^
Caucasian, *n* (%)	459 (93%)	329 (93%)	130 (94%)	0.90^b^
BMI, median (IQR)	28.6 (25.7, 31.7)	28.7 (25.6, 31.8)	28.4 (26.0, 31.3)	0.97^a^
Cardiac risk factors and medical history				
Family history of CAD, *n* (%)	214 (47%)	146 (45%)	68 (52%)	0.14^b^
Dyslipidaemia, *n* (%)	352 (72%)	245 (69%)	107 (77%)	0.094^b^
Previous MI, *n* (%)	63 (13%)	43 (12%)	20 (14%)	0.49^b^
Previous stroke, *n* (%)	18 (3.7%)	11 (3.1%)	7 (5.0%)	0.31^b^
Hypertension, *n* (%)	307 (62%)	213 (60%)	94 (68%)	0.13^b^
Diabetes mellitus, *n* (%)	106 (22%)	80 (23%)	26 (19%)	0.34^b^
History of HD, *n* (%)	485 (99%)	347 (99%)	138 (100%)	0.33^c^
History of CAD, *n* (%)	483 (98%)	345 (98%)	138 (99%)	0.46^c^
Previous TIA, *n* (%)	12 (2.4%)	9 (2.5%)	3 (2.2%)	>0.99^c^
Previous cancer, *n* (%)	85 (18%)	60 (17%)	25 (19%)	0.70^b^
Current smoker, *n* (%)	173 (35%)	120 (34%)	53 (38%)	0.59^b^
Previous PCI, *n* (%)	57 (12%)	37 (11%)	20 (14%)	0.22^b^
Previous CABG, *n* (%)	11 (2.2%)	7 (2.0%)	4 (2.9%)	0.51^c^
PCI presentation				
Culprit artery				
LAD (%)	327 (69)	245 (73)	82 (59%)	0.005
RCA (%)	98 (21)	55 (16)	43 (31)	0.001
CX (%)	34 (7)	24 (7)	10 (7)	0.90
Ischaemic time, minutes, median (IQR)	151 (108, 245)	159 (111, 273)	140 (100, 194)	0.011^a^
Door to balloon, minutes, median (IQR)	64 (49, 83)	64 (49, 82)	66 (48, 88)	0.76^a^
ST-elevation pre-procedure, mm, median (IQR)	2.50 (2.00, 4.00)	3.00 (2.00, 4.00)	2.50 (2.00, 3.50)	0.026^a^
Killip class, *n* (%)				0.81^c^
0	1 (0.2%)	1 (0.3%)	0 (0%)	
1	442 (90%)	317 (90%)	125 (90%)	
2	11 (2.2%)	9 (2.5%)	2 (1.4%)	
3	7 (1.4%)	4 (1.1%)	3 (2.2%)	
4	31 (6.3%)	22 (6.2%)	9 (6.5%)	
Cardiogenic shock, *n* (%)				0.99^b^
Pre-PCI	33 (6.7%)	24 (6.8%)	9 (6.5%)	
Post-PCI	22 (4.5%)	16 (4.5%)	6 (4.3%)	
TIMI flow pre, *n* (%)				0.003^b^
0	321 (66%)	247 (71%)	74 (54%)	
1	35 (7.2%)	23 (6.6%)	12 (8.8%)	
2	66 (14%)	41 (12%)	25 (18%)	
3	61 (13%)	35 (10%)	26 (19%)	
TIMI flow post, *n* (%)				0.95^c^
0	7 (1.4%)	5 (1.4%)	2 (1.5%)	
1	1 (0.2%)	1 (0.3%)	0 (0%)	
2	17 (3.5%)	13 (3.8%)	4 (2.9%)	
3	458 (95%)	327 (95%)	131 (96%)	
Cardiac MRI indices				
LVEDVI (mL/m^2^), median (IQR)	78 (66, 89)	80 (67, 91)	75 (65, 86)	0.026^a^
LVESVI, (mL/m^2^), median (IQR)	37 (28, 48)	39 (30, 51)	30 (24, 39)	<0.001^a^
LVEF (%), median (IQR)	50 (42, 59)	48 (40, 55)	58 (49, 65)	<0.001^a^
LV mass (g), median (IQR)	143 (119, 170)	146 (122, 172)	134 (112, 161)	0.004^a^
Infarct size (g), mean, SD, (*n*)		44 ± 28 (83)	27.6 ± 20.1 (73)	0.001
MVO mass (g), mean, SD		16.2 ± 19.2		
Persistent ST-elevation post-proc., *n* (%)	224 (46%)	187 (54%)	37 (27%)	<0.001^b^
LVEF (echo) at PCI, median (IQR)	40 (32, 50)	40 (32, 50)	45 (35, 55)	0.005^a^
IABP, *n* (%)	59 (12%)	44 (12%)	15 (11%)	0.61^b^
Laboratory values				
CKMB peak, median (IQR)	134 (60, 273)	174 (89, 350)	70 (32, 138)	<0.001^a^
Peak creatinine, median (IQR)	1.03 (0.88, 1.20)	1.03 (0.89, 1.20)	1.03 (0.85, 1.23)	0.66^a^
Baseline haemoglobin, median (IQR)	14.60 (13.50, 15.70)	14.60 (13.47, 15.70)	14.70 (13.60, 15.70)	0.79^a^
Total cholesterol, median (IQR)	165 (138, 192)	165 (138, 190)	165 (138, 196)	0.60^a^
Triglycerides, median (IQR)	118 (84, 162)	120 (86, 160)	116 (80, 166)	0.76^a^
HDL, median (IQR)	37 (32, 45)	37 (32, 45)	38 (32, 46)	0.36^a^
LDL, median (IQR)	99 (78, 123)	99 (78, 122)	100 (78, 126)	0.70^a^
Length of stay, days, median (IQR)	3 (2, 5)	3 (2, 5)	3 (2, 4)	0.010^a^
In-hospital death, *n* (%)	8 (1.6%)	6 (1.7%)	2 (1.4%)	>0.99^c^

BMI, body mass index; CAD, coronary artery disease; MI, myocardial infarction; HD, haemodialysis; TIA, transient ischaemic attack; PCI, percutaneous coronary intervention; CABG, coronary artery bypass surgery; LAD, left anterior descending; RCA, right coronary artery; CX, circumflex; LVEDVI, left ventricular end-diastolic volume index; LVESI, left ventricular end-systolic index; LVEF, left ventricular ejection fraction; MVO, microvascular obstruction; IABP, intra-aortic balloon pump; CK, creatine kinase; HDL, high density lipoprotein; LDL, low density lipoprotein.

^a^Wilcoxon rank sum test.

^b^Pearson’s χ^2^ test.

^c^Fisher’s exact test.

During long-term follow-up which averaged 8.4 ± 3.8 years, a total of 56 patients with MVO died compared to 18 patients without MVO (*Table 2*). Patients with MVO died sooner following their STEMI (5.9 vs. 7.8 years) and were more likely to die from cardiovascular causes such as progressive heart failure or sudden cardiac death.

**Table 2:** Cause of death for patients with and without microvascular obstruction

Cause of death	Cardiovascular CHF Cardiac arrest MI Stroke/ICH	Neurological Dementia ICH	Sepsis	Pulmonary Pneumonia Respiratory failure COVID-ARDS	Cancer	Natural causes	Time from STEMI to death (years)
MVO+ (*n* = 56)	25^a^	5	6	7	9	4	5.9 ± 4
MVO− (*n* = 18)	2	0	0	3	7	6	7.8 ± 4

CHF, congestive heart failure; MI, myocardial infarction; ICH, intracranial haemorrhage; ARDS, adult respiratory distress syndrome.

^a^ *P* = 0.004 Fisher’s exact test.

## Discussion

Previous studies have identified the presence of MVO following STEMI to be associated with increased hospitalization for heart failure and mortality in the first 1 to 2 years following presentation.^1,2^ van Kranenburg *et al*.^1^ observed that MVO was associated with cardiac death when adjusted for age and LVEF in a meta-analysis of 1025 patients enrolled between 1999 and 2008. de Waha *et al*.^2^ demonstrated that the presence of MVO was associated with an increase in all-cause death in the first year of follow-up and that the rate of mortality increased with increasing MVO mass in seven randomized primary PCI trials (*n* = 1688). Only one study has previously examined the long-term effects of MVO on mortality. Symons *et al*.^3^ observed that late MVO was a strong independent predictor of the composite of all-cause death and HF hospitalization in a cohort of 810 patients with STEMI followed for a median duration of 5.5 years. They observed a total of 23 cardiac deaths and 16 non-cardiac deaths from a research consortium of six sites but did not differentiate mortality outcomes by the presence or absence of MVO. In contrast to their study measuring late MVO, our analysis measured early MVO. Although late MVO is felt to provide better long-term prognostic information on major adverse cardiovascular events and mortality compared to first-pass and early MVO measurements,^7^ our results provide clear evidence that early MVO can also demonstrate a strong relationship to mortality. The increased sensitivity of early MVO measurements^7^ provides confidence that the patients stipulated as not having MVO were truly free of MVO in our cohort.

The relatively higher mortality we observed may be secondary to the greater infarct size and MVO mass in our cohort and our inclusion of cardiogenic shock patients. Our study adds several new findings to these previous observations regarding the relationship between MVO and mortality. Although we confirm that the presence of early MVO is associated with greater infarct size and reduced LV function, it is the first report to describe the causes of death over the longest duration of follow-up between STEMI patients with and without MVO. Here, we demonstrate that the vast majority of patients with MVO die from cardiovascular and cerebrovascular causes. In contrast, STEMI patients without MVO live longer and rarely die from cardiovascular causes. This may suggest that the presence of MVO following STEMI in addition to reflecting greater myocardial damage may be a surrogate marker for greater atherosclerotic disease burden and/or microvascular dysfunction which could account for its disparate effect on mortality and heart failure despite occupying such a small region of the infarct area. We were unable to measure intramyocardial haemorrhage (IMH) in our cohort but we suspect that future studies will demonstrate a powerful role for IMH in mediating heart failure and mortality following STEMI that supersedes MVO.^8^

### Limitations

This clinical study has several limitations. First, this study was executed at a single health care centre in Minneapolis, which has limited diversity and the patient population and may not be representative of the general STEMI population. Secondly, cardiac MRI prior to discharge was not done routinely for all STEMI patients but was at the discretion of the treating physician resulting in a smaller sample size and possible selection bias and accounts for the higher percentage of patients with MVO. Overall, our small cohort size was a limiting factor and should be considered hypothesis-generating, as a more robust dataset would provide greater insight into our mortality observations.

## Data Availability

The data underlying this article will be shared on reasonable request to the corresponding author.

## Lead author biography

Giselle Fisher is a second-year medical student at the Perelman School of Medicine, University of Pennsylvania. She was selected for the Minneapolis Heart Institute Foundation’s Clinical Research Internship where she worked with Dr Jay Traverse. Clinically, she is interested in chronic disease management in underserved populations, preventive cardiology, and coronary artery disease. Her current research interests include microvascular dysfunction and the interplay between lipid metabolism and cardiovascular disease.
